# Political Systems Affect Mobile and Sessile Species Diversity – A Legacy from the Post-WWII Period

**DOI:** 10.1371/journal.pone.0103367

**Published:** 2014-08-01

**Authors:** Sara A. O. Cousins, Mitja Kaligarič, Branko Bakan, Regina Lindborg

**Affiliations:** 1 Landscape Ecology, Department of Physical Geography and Quaternary Geology, Stockholm University, Stockholm, Sweden; 2 University of Maribor, Biology Department, Faculty of Natural Sciences and Mathematics, Maribor, Slovenia; 3 Faculty of Agriculture and Life Sciences, University of Maribor, Pivola 10, Hoče, Slovenia; University of Kent, United Kingdom

## Abstract

Political ideologies, policies and economy affect land use which in turn may affect biodiversity patterns and future conservation targets. However, few studies have investigated biodiversity in landscapes with similar physical properties but governed by different political systems. Here we investigate land use and biodiversity patterns, and number and composition of birds and plants, in the borderland of Austria, Slovenia and Hungary. It is a physically uniform landscape but managed differently during the last 70 years as a consequence of the political “map” of Europe after World War I and II. We used a historical map from 1910 and satellite data to delineate land use within three 10-kilometre transects starting from the point where the three countries meet. There was a clear difference between countries detectable in current biodiversity patterns, which relates to land use history. Mobile species richness was associated with current land use whereas diversity of sessile species was more associated with past land use. Heterogeneous landscapes were positively and forest cover was negatively correlated to bird species richness. Our results provide insights into why landscape history is important to understand present and future biodiversity patterns, which is crucial for designing policies and conservation strategies across the world.

## Introduction

It is increasingly recognized that conservation biology should have a “landscape perspective” [1]–[4]. This is generally understood in a spatial context when considering targets for conservation, but a temporal dimension of the landscape is also necessary to understand effects of delayed species responses. This is however rarely considered. Land use change, either by intensification or abandonment, is one of the main drivers causing deterioration of species-richness across the world [5]. Land use and vegetation structure and composition, commonly used as explanatory factor for biodiversity patterns, is to a large extent outcomes of political and socio-economic decisions or constraints. However, the same driving forces may lead to different effects depending on the physical landscape [6], [7], e.g. differences in soil fertility, topography or water availability. Land use effects on biodiversity are highly debated topics, especially in conservation research [8], [9], because a lack of spatially explicit historical biodiversity data. However, there is a consensus that the decline in traditional agriculture often has negative effects on biodiversity, as the low intensive utilisation of grasslands and forests in the past has been a prerequisite for much of the high small-scale species richness found in the rural landscape of Europe today [10]–[12]. Species' richness, abundance and composition may respond directly to land use changes but a delayed response has been detected in several studies [13], [14]. Such responses often differ depending on organism group, where many mobile organisms respond more quickly to landscape change compared to long-lived sessile organism [15], [16].

Despite the increased awareness of social-ecological linkages [17] in conservation, few studies have used large scale *in situ* experimental designs to analyze direct or indirect effects of non-ecological drivers on biodiversity patterns. One reason is the difficulty to find suitable study systems as the divisions into countries or regions often are a result of underlying physical landscape differences [18]. Furthermore, magnitude and timing of intensifications or abandonment is also constrained by physical properties at local or regional scales. For example, Cousins [19] found that areas with a larger proportion of clayey soils changed towards intensive crop-production earlier than areas with smaller proportions of clayey soil. Landscapes on more marginal soils or locations have shown a tendency to be abandoned and afforested [20], [21]. However, because of geopolitical reasons, during the last 100 years, there are regions all over the world that have been divided without considering physical landscape divisions or uniformities. Beside former colonies in Africa and Asia some recent examples are Korea and New Guinea. Considerable differences between Eastern and Western Europe, regarding bird and plant diversity, and forestry, have previously been highlighted by several authors [22]–[24], but are rarely addressed in the design and interpretation of research or policy, but see [25], [26]. In Europe, the division between socialist and non-socialist states since WWII, and the recent agenda to privatize or re-privatize the former socialist economies, has especially affected agricultural systems. During the socialist era, there was a widespread collectivization resulting in few but large farming units, contrasted by many small co-existing non-industrial farms outside the industrial food production [27]. Also in Western Europe, driven by market economy, the majority of traditional small scale farms have disappeared due to intensified and specialized agriculture [28], [29] or abandonment and afforestation [19].

In this study, we compare current biodiversity patterns in a physically uniform area in the borderland where three countries meet, Austria, Hungary and Slovenia. The historical management in this part of Europe was dominated by small-scale traditional farming 100 years ago when it was part of the same state; the Austrian-Hungarian Empire. Although the area was divided into three countries after 1921, the economic system (market oriented traditional agriculture) remained more or less the same until 1945. After 1945 the difference between countries became prominent, due to changed political systems. In Austria almost all land were privately owned, driven by a free market Western economic policy, whereupon fields became more intensively used and larger in general. Slovenia (part of Yugoslavia) became a pronounced socialist market economy. Large farms on the lowlands were confiscated and collectivised, but in the hilly remote region near the borders to Hungary and Austria the majority of the land remained private and fairly small-scaled. In contrast, the command economy in Hungary eroded the traditional farming system through collectivisation of farms, where former landowners became employed as workers in big state-owned farms. Thus, both free-market economy and collectivization resulted in larger and more intensively used farms where the physical landscape made it possible. Along the Iron Curtain (i.e. here the border to Austria and Slovenia) there was a policy of depopulation. Since the fall of former Yugoslavia and the Eastern Block in the 1990s, Hungary and Slovenia has moved to a free market economy and are both part of the European Union since 2004, whereas Austria became a member state in 1995. Today all nations are part of EU and the “borderless” Schengen region. It is important to note that after the socialist era, no quick, abrupt or radical changes in land use or land ownership occurred in the studied region.

Our primary focus is on landscape matrix effects (as a consequence of land use change) on biodiversity. The rationale behind this study is that the different political systems during the last 70 years will be reflected in the land use and hence also in biodiversity patterns [13], here analysed by using mobile (birds) and sessile (plants) species. We hypothesize that traditionally managed landscape has highest biodiversity and intensified agricultural landscape has the lowest biodiversity [29]–[31] but see [32]. Furthermore, mobile organisms will be more associated with current land use structure than less mobile organisms [15], exposing a legacy from the land use prior to the post-war era.

## Methods

### Land cover

**Figure 1:**
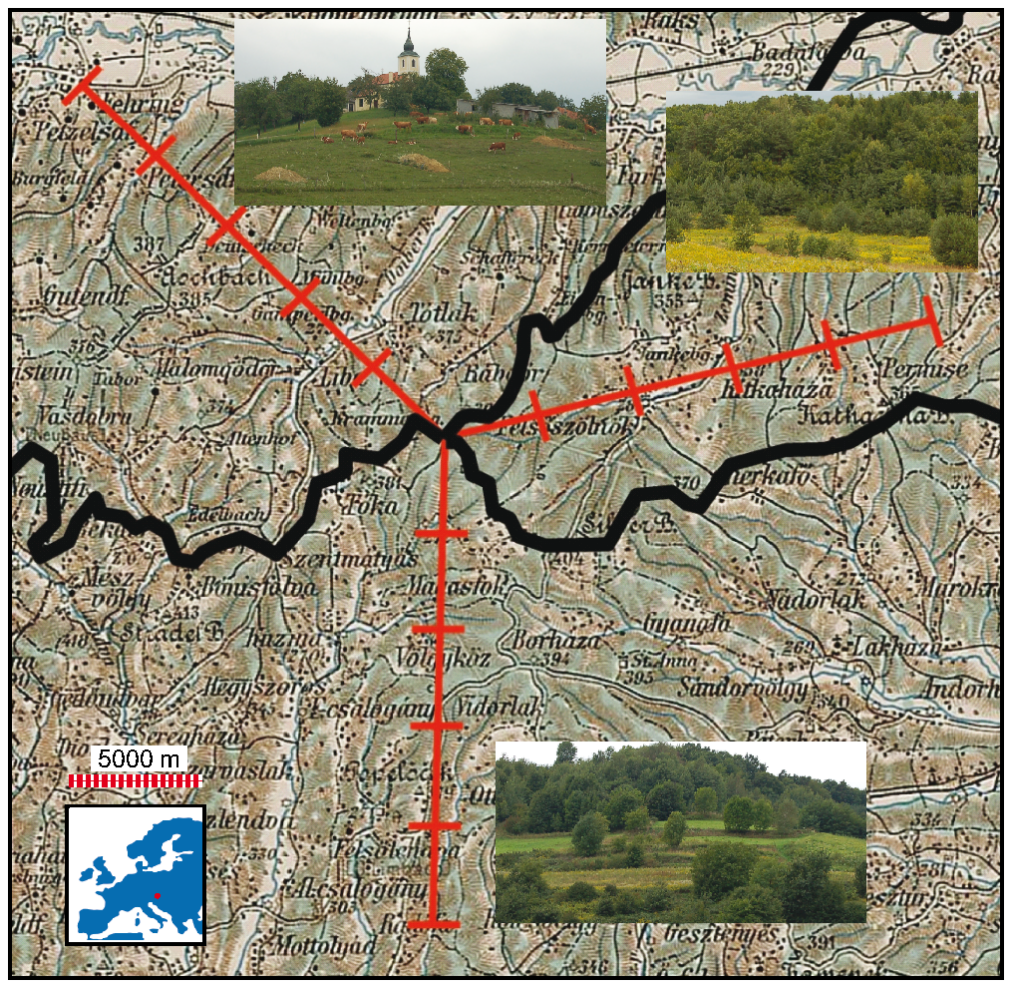
Sampling design for investigating bird and plant diversity in three bordering landscapes in Austria, Slovenia and Hungary. Birds and plants were inventoried in 2-kilometers long cross-transects along three 10 kilometer long main transects radiating out from the point where Austria, Slovenia and Hungary meet today. The investigated landscapes belong to the Trilateral Park: Raab (Austria), Goričko (Slovenia) and Őrség (Hungary). The historical land cover map is from 1910, under the Austrian-Hungarian Empire, showing the study area with the present-day borders (black) superimposed. Green areas are forested land and pink areas are arable land.

Because mobile organisms are expected to respond more quickly to landscape change compared to long-lived sessile organism we chose to investigate both birds and plants. Methods were chosen to best fit the mobility of the organisms i.e. transect mapping for birds and plot inventory for plants. Birds and plants were inventoried in 2-kilometers long cross-transects along three 10 kilometre long main transects radiating out from the point (46,869° N; 16,114° E) where Austria, Slovenia and Hungary meet (Fig. 1). Each main transect ends in Austria at 46,556 N; 16,116 E, in Hungary at 46,532 N; 16,143 E and in Slovenia at 46,465 N; 16,637 E. The climate is moderate continental or sub-Pannonic, with relatively dry winters and with an average annual rainfall of 900 mm. Mean temperature in January is −2°C and in July 19°C. Geologic substrates are mainly tertiary sediments, which forms a soft hilly landscape of sandy-acid soils with networks of running fresh water. The landscape is a mixture of forest and open areas with small farms scattered along hilltops. The investigated landscapes belong to the Trilateral Park: Raab (Austria), Goričko (Slovenia) and Őrség (Hungary). Raab is a nature park (established 1997) aiming to preserve traditional landscapes, Goričko is a Natura 2000 area (established 2002) with the goal to keep traditional and extensive small-scale farming, and Őrség National Park and Natura 2000 area was established 2004 to promote wild-life and tourism and preserve the unique Oak-Pine forests.

Hereafter these different landscapes will be referred to as Raab (Austria), Goričko (Slovenia) and Őrség (Hungary) although it should be noted that the study does not encompass the whole of each park. We used a historical map from 1910 (Fig. 1) from the Austrian-Hungarian Empire (3rd Military Mapping Survey of Austria-Hungary, sheet “Szombathely”), to estimate the relationship between open and forest land in the past. Unfortunately it was not possible to carry out any detailed analyses on land cover composition because of low thematic resolution. To link current biodiversity to land use, we calculated current land use in the study region using CORINE land-cover data from 2000 with a resolution of 50×50 m. We used each 10-km transect (Fig. 1) with sample cross-section width, i.e. 10×2 km, to calculate percentages of different land-cover classes from CORINE in a geographical information system (GIS). Maps of topography and soils were cross-checked to detect dissimilarities between the different transects.

### Field survey

A field inventory of birds and plants was designed to capture the differences in land use as well as biodiversity patterns within the countries (File S1). Bird diversity was investigated by slowly walking along the cross-section transect and noting all birds seen or heard following the transect mapping method [33]. All cross-transects were visited twice during the breeding season (spring and late summer), which resulted in 5 samples for each country. Bird classifications follows the nomenclature by Geister [34] and Svensson & Mullarney [35].

The plant inventory was conducted during the field-season 2010. Along the cross-section transects 21 sampling points were placed evenly every 50 meters, in total 105 sampling points in each country. First, all vascular plants found within a 2×2 m square were noted, and then all additional plants found in a circle with radius of 10 m around the plot were added. In addition, we noted the main land-cover type for each 10 m radius plot: grassland (grazed or mown), forest, field (for crop-production) and ruderal or urban surfaces land (for example house, road, playground), which is hereafter referred to as ruderal. For each sampling point in forest the age of trees were categorized as: >30 years old, between 15–30 years or <15 years. Plant nomenclature followed Martinčič et al. [36]. No specific permissions were required for any of the field studies. Only observational studies were performed without interference of plants or birds.

### Statistical analysis

Number of bird and plant species in relation to nation and land use type was analysed in separate ANOVAs. To examine how the number of bird species was affected by land use type we used the proportion of present day forest-cover in an ANCOVA, using forest cover as explanatory variable and nationality as covariate. “Nationality” (Austria, Hungary, and Slovenia) is arbitrarily used here to reflect past land use history i.e. past political system. To investigate differences in plant composition, nationality and each cross transect distance from the border were predictor variables. We used the model Generalized Estimating Equations (GEE) [37] which allows for terms specifying autocorrelation and are well suited for evaluating landscape processes. First we did a Principal Coordinates Analysis (PCoA), based on dissimilarity (Bray-Curtis) of plant species at the 10 m scale, using the first three axes. We would expect similarity to decrease with distance from where the transects meet, both due to distance in itself and because of differences in land use. Models including both predictor variables transect and nationality, as well as a model with nationality only, were tested against the null model (only including random effects). The significance of each model was tested with likelihood ratio test and post hoc test to separate each variable within the predictors. Data deviating from a normal distribution was log10 transformation and all numbers were increased by one before analysis. The statistical software R 2.13.0 was used for the analyses using the *geepack* package for GEE modeling [37].

## Results

### Land use change

**Figure 2:**
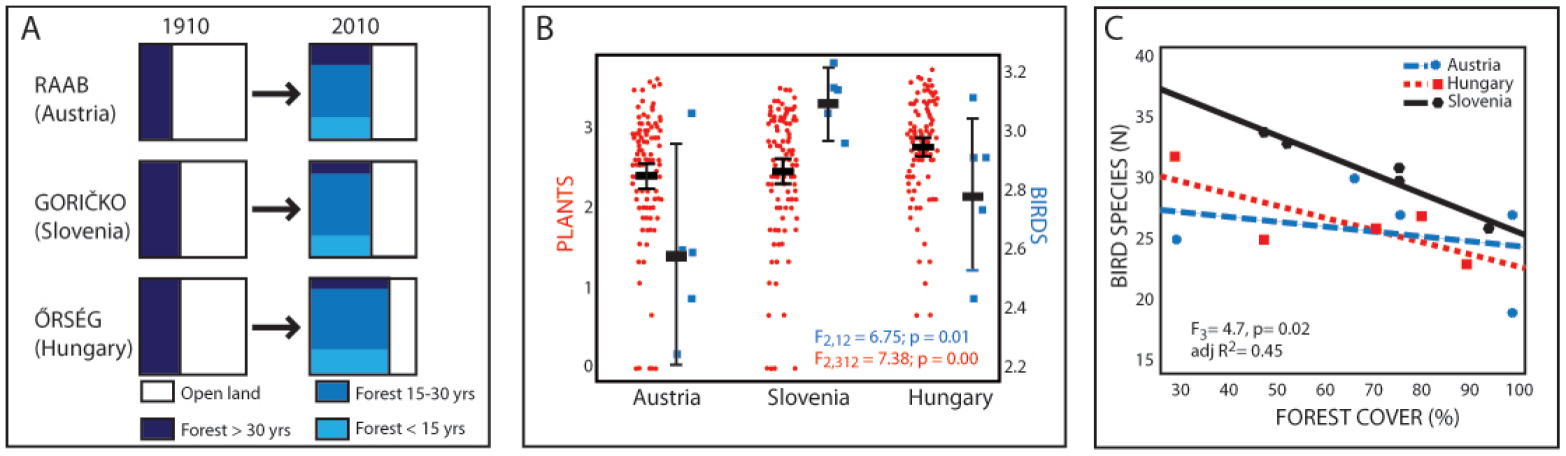
100 year of land cover change and present-day plant and bird diversity in the border landscapes in Austria, Slovenia and Hungary. (A) Open and forest land cover within the transects between 1910 to 2010. The map from 1910 is thematically coarser so it is not possible to separate the difference in forest age. (B) Mean numbers of plants (red circles) and birds (blue squares) from plots along transects in each country. (C) The percentage of forest cover along each cross-transect and the relationship to number of bird species. There were 5 cross-transects investigated in each country.

In 1910, the landscape along each transect was dominated by open agricultural land with a forest cover between 30–39%; a landscape composition that today is inverted with forests covering between 57–74% of the landscapes (Fig 2). Based on the plot surveys, Raab (Austria) has the highest current percentage (23%) of forest older than 30 years, whereas 65% of the forests in Goričko (Slovenia) and 67% in Őrség (Hungary) are between 15 and 30 years old. Young forests (<30 years) are primarily on former arable fields or grasslands. In the investigated landscapes grasslands are few and arable fields even more rare: 11% and 4% Raab (Austria), 19% and 1% Őrség (Hungary) and 15% and 9% Goričko (Slovenia) for grassland and fields, respectively.

### Bird diversity patterns

We found 53 different birds species in total (Table 1; File S1). The bird species composition was 40% in total overlap between the three countries, and 55% to 58% when comparing countries pair wise. There was a significant difference between countries (F_2,12_ = 6.747, p = 0.0108) (Fig. 2), where Goričko (Slovenia) had significantly higher diversity of birds than Raab (Austria) (Tukey HSD, p<0.0087), but there were no significant difference between Őrség (Hungary) and Raab (Austria) or Goričko (Slovenia) (Tukey HSD, p = 0.37 and p = 0.1 respectively). When associating different bird species to habitat most birds were classified as forest species; Goričko (Slovenia) had 28% (29 species), Őrség (Hungary) 25% (27 sp.) and Raab (Austria) 24% (28 sp.). Only a few percent of the bird species were associated to open grassland habitats: Raab (5%), Goričko (15%) and Őrség (7%). Number of birds was clearly related to forest cover in each transect, with a significant difference between frequencies of birds found in transects depending on forest cover and land use history (i.e. nationality) as bird diversity declined with an increase in forest cover (Fig. 2). Although the trend is similar for all countries it is only significant for Slovenia (F_3_ = 4.7, p = 0.02, adjusted R2 = 0.45, ANCOVA).

**Table 1:** Species richness of plants and birds based on surveys along three 10 km long transects in Hungary, Slovenia and Austria.

	Austria		Slovenia		Hungary	
	plants	birds	plants	birds	plants	birds
Total (n)	256	43	281	47	299	42
2×2 plot (SD)	7.2 (5.3)	-	8.5 (5.8)	-	9.3 (6.6)	-
10 m radius (SD)	14.8 (9.7)	-	15.6 (9.4)	-	19.7 (10.4)	-
Cross-transect (SD)	-	13.4 (4.6)	-	21.8 (2.3)	-	16.0 (3.1)

Total (n) is the total number of different species found in each country. Richness of plants is recorded at two different spatial scales, 2×2 m and 10×10 m (105 plots in each country), and bird richness is recorded per cross-transect (5 transects in each country). Grassland/forest species is the number of specialists species for grassland and forest habitats found. (-) indicate not applicable.

### Plant diversity patterns

In total, we found 407 vascular plant species with relatively few endangered plant species, according to (separately considered) national Red Lists: Raab (Austria) 7% (18 sp.), Goričko (Slovenia) 1% (3 sp.) and Őrség (Hungary) 5% (14 sp.) (File S1). Only 180 out of 407 species occurred in all the three countries. There was a significant difference in plant species richness among countries at the larger (314 m^2^) scale. Őrség (Hungary) had more plant species than Goričko (Slovenia) and Raab (Austria) (F_2,312_ = 7.38; p = 0.0007) (Fig. 2). At the smaller scale (4 m^2^) no significant difference could be detected for plant richness between countries (p>0.11; Table 1). Plots in grassland habitats had significantly higher plant richness compared to all other habitats, irrespective of country (F_3,310_ = 14.9; p<0.0001).

**Figure 3:**
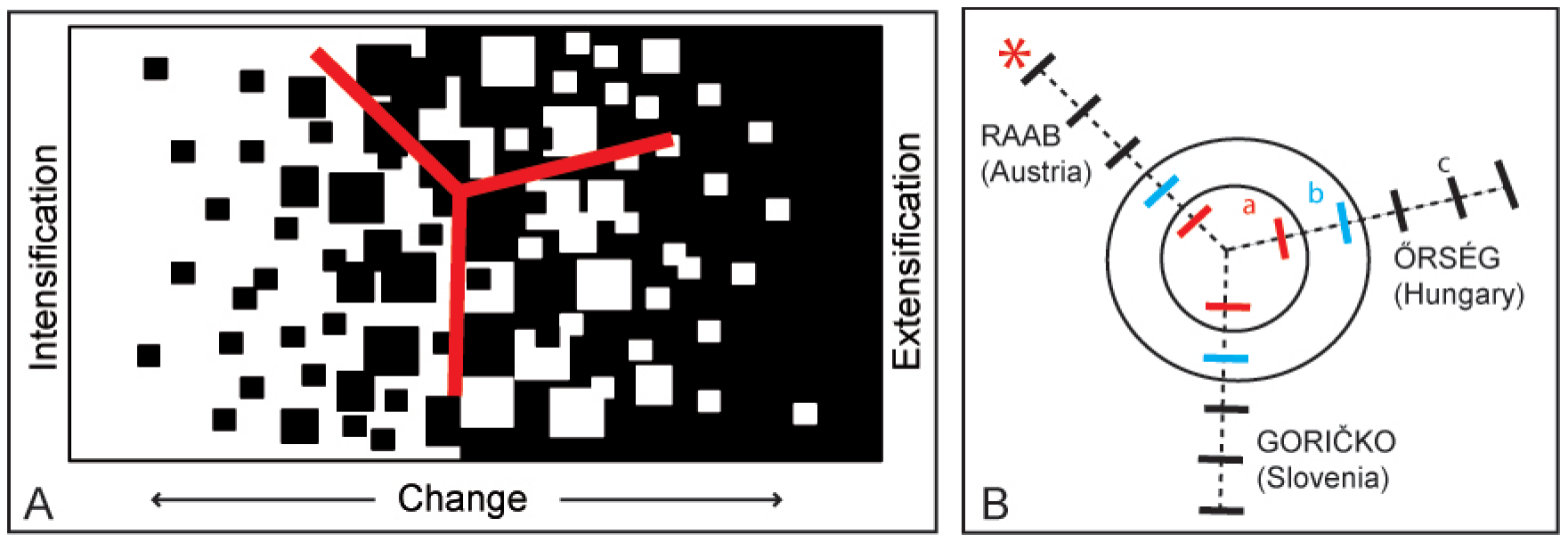
A conceptual model (A) shows how the three areas have either changed because of intensification (Austria) or extensification (Hungary) or remained more or less status quo (Slovenia). Similarity in plant species composition along cross-transects (B) spanning out from the point where the three countries meet. Significant difference in composition is marked with different letters a, b or c. An * marks the significant difference in species composition between countries.

In the GEE analyses, using distance of cross-transects and country as predictive variables on plant composition, we found that distance from the border clearly affected plant community structure were there also was a clear effect of nationality (likelihood ratio test: χ^2^ = 19.1, DF = 4, p = 0.0008). The post hoc test showed that the species composition in first cross-transect (closest to the border, Fig. 3b) is separated from the second which are both separate from the third to fifth. Furthermore the species composition in Raab (Austria) is different compared to both Őrség (Hungary) and Goričko (Slovenia).

## Discussion

To understand the processes behind, and to predict, biodiversity patterns we need to analyse landscape history [38]. Unfortunately there are hardly any records of historical species diversity patterns, making it impossible to analyse direct effects of landscape changes on species richness and composition but see [21], [39]. However, historical maps together with landscape change trajectories have been used to indirectly analyse how biodiversity patterns are affected [10], [40], [41]. Here we show, by using 100 year old landscape data, that the difference in historical political systems during the last 70 years can be detected on present-day species diversity patterns. Hundred years ago the landscape along the investigated transects was dominated by open agricultural land with a forest cover between 30–40%, whereas it is today inverted with forests covering between 60–70%. Many arable fields and grasslands have become afforested, particularly in Hungary. The current landscape composition is fairly similar in the three landscapes, considering the percentage of forest cover to open land (Fig. 2a) although land use changes and different conservation strategies have resulted in clear differences in species composition (Fig. 2b). In the Hungarian Őrség, rewilding [42], [43] has led to increasing forest area, whereas Goričko (Slovenia) is more similar to the traditional landscape before the changes after 1945. Despite Austria and Slovenia having similarities in conservation goals, higher biodiversity of both plants and birds was noted in Slovenia compared to Austria. These biodiversity patterns could be indirectly linked to differences in political system and economic drivers, where the market oriented agriculture in Austria, compared to the more subsistence agriculture in the area in Slovenia during the whole period, has resulted in an intensification of agriculture through the EU Common Agricultural Policy (CAP) funding structures.

The distribution pattern of mobile and sessile organisms varied depending on country (i.e. nationality). The more traditional landscape in Goričko (Slovenia) had significantly higher diversity of birds compared to the more intensively managed Raab (Austria), but there were no difference between the Hungarian landscape and the other two. The bird composition similarity among areas was low compared to other studies [44], [45], especially considering the high mobility of birds that can move across national borders for nesting and feeding. The frequencies of birds found in transects depended on forest cover, where bird diversity declined with increasing forest cover. Small-scale heterogeneity, i.e. structurally more complex landscapes, in contemporary landscapes favour bird species richness, whilst a denser forest-cover has a negative effect on bird diversity. Several models suggest that more wildlife-friendly farming and heterogeneous landscapes, with many small natural or semi-natural habitats, help to support a relative high diversity compared to large scale farming and commercial forestry which is negative for biodiversity [46], [47].

The highest plant species richness was found in Őrség (Hungary), with many typical grassland plant species, despite being primarily conserved and managed as forest. Prior to the Eastern Bloc policy to depopulate and reforest the area it was managed as a traditional agricultural landscape with many orchards and grasslands, and today remnant grassland communities intermingle with colonizing forest species. Particularly long-lived organisms, like plants, may survive as remnant populations for a long time after management has ceased [48], creating a so-called extinction debt [14], [15], [40], [49]. Cousins [50] estimated a threshold for extinction debt in plant communities in grasslands to be settled after around 70 years, in Northern Europe. Many rural ecosystems have a long history of co-evolution with human management and today the survival of many species depends on the maintenance of low intensity farmland practices [51]–[53]. A historical dimension is hence a necessary complement to the spatial conservation perspective, particularly in landscapes where biodiversity is associated to traditional management. Here, we expect that many plant species associated to the remnant grassland habitats will disappear in Őrség (Hungary). However, other organisms might benefit and those associated to forest habitats should increase with succession of young forests [54]. We envisage based on the past and current trajectories that in the future the rewilding in Hungary will lead to that the legacy from past grassland composition will disappear in favour of forest biodiversity. The traditional landscape in Austria will probably remain fairly stable, but for the traditional landscape in Slovenia to remain, subsidies are needed and a functioning infrastructure to increase retailing of farm products. Thus, the long-term legacies from the pre-war landscape will disappear slowly and differences in plant diversity patterns become even more pronounced in the future also close to the border at a local scale, despite that there formally are no borders any more.

Although we cannot explain the direct causes for the differences in biodiversity patterns, due to possible co-variation of unknown environmental variables, the study area is relatively small and the abiotic conditions similar, i.e. bedrock and soil types, topography, climate; which strongly infers that the effects are a consequence of land use change, linked to past political systems. Both intensification and abandonment are clear results of political systems during the last 70 years. Similar effects have been observed also within the countries that used to lie within the Eastern Bloc where the different political systems established after the collapse of Soviet Union. For example, a comparison between e.g. Poland, as a EU country, to Russia and Belarus showed different trajectories of landscape transformations caused by agricultural abandonment [55]. Studies from areas, not confounded by underlying abiotic landscape differences, but driven by different political and economical policies can further disentangle how biodiversity patterns may change in the future [25], [26]. There are several other comparable political divisions outside Europe that potentially can be used as experimental sites for investigating landscape history's effect on biodiversity and conservation.

In this study we give an *in situ* example of how national political priorities for social structure and economy may drive regional changes in land use that affects species diversity and composition. As expected, traditional agricultural landscape in Slovenia had the highest diversity but only for birds. Heterogeneity (here traditional agricultural landscape) at a landscape scale is expected to favour also plants, but we found that the heterogeneity in time (new land uses superimposed on former land use) created higher plant richness (Fig. 3a and 3b). Thus the hypothesis that mobile species richness is more associated to current land use and many sessile species are more associated to past land use is confirmed, as shown for plant diversity and composition in the Hungarian transect. We stress that awareness of how political and economical decisions directly or indirectly affect land use and biodiversity is crucial information, not only for the managers of these particular conservation areas, but also for designing sustainable policies and conservation strategies across the world.

## Supporting Information

File S1 **Species data.** Plants and birds species found in Austria (AUS), Slovenia (SLO) and Hungary (HUN). + indicates occurrence during the field survey in respectively country. If a plant specie occurs on the National Red List (Anonymous [56] for Slovenia, Gergely [57] for Hungary and Niklfeld [58] for Austria) it is indicated with a 1. The typical habitats for plants were classified as forest (F), ruderal (R) and grassland (G) on the basis of the local flora monograph [59]. The birds were classified; [43] into species typical for forest (F), open and grassland habitats (O), settlements (S) or mixed habitats (M) including both open and forest landscape habitats. Bird abundance was classified as very common (vC), common (C) or rare (R) and breeding status as resident (rB), migratory (mB) and possible breeding species (rB?). Total numbers of plant and bird taxa found were 407 and 53 respectively.
(DOCX)
