# Supplementary material for: Political Systems Affect Mobile and Sessile Species Diversity – A Legacy from the Post-WWII Period
Source: PLoS One. 2014 Aug 1;9(8):e103367. doi: 10.1371/journal.pone.0103367 (PMC4118865; doi:10.1371/journal.pone.0103367)
Supplement: File S1 — Species data. Plants and birds species found in Austria (AUS), Slovenia (SLO) and Hungary (HUN). + indicates occurrence during the field survey in respectively country. If a plant specie occurs on the National Red List (Anonymous [56] for Slovenia, Gergely [57] for Hungary and Niklfeld [58] for Austria) it is indicated with a 1. The typical habitats for plants were classified as forest (F), ruderal (R) and grassland (G) on the basis of the local flora monograph [59]. The birds were classified; [43] into species typical for forest (F), open and grassland habitats (O), settlements (S) or mixed habitats (M) including both open and forest landscape habitats. Bird abundance was classified as very common (vC), common (C) or rare (R) and breeding status as resident (rB), migratory (mB) and possible breeding species (rB?). Total numbers of plant and bird taxa found were 407 and 53 respectively. (DOCX) [file pone.0103367.s001.docx]

|  | Occurrence | | | National Red List | | | Typical |
| --- | --- | --- | --- | --- | --- | --- | --- |
| Plant taxa list | AUS | SLO | HUN | AUS | SLO | HUN | Habitat |
| Abies alba |  | + |  |  |  |  | F |
| Abutilon theophrasti |  | + |  |  |  |  | R |
| Acer campestre | + | + | + |  |  |  | F |
| Acer platanoides |  |  | + |  |  |  | F |
| Acer pseudoplatanus | + | + | + |  |  |  | F |
| Achillea millefolium agg. | + | + | + |  |  |  | G |
| Achillea ptarmica |  |  | + | 1 |  | 1 | G |
| Actaea spicata | + | + | + |  |  |  | F |
| Aegopodium podagraria | + | + | + |  |  |  | F |
| Agrimonia eupatoria | + | + | + |  |  |  | G |
| Agrostis canina | + | + | + |  |  |  | G |
| Agrostis stolonifera | + |  |  |  |  |  | G |
| Agrostis tenuis | + | + | + |  |  |  | G |
| Ajuga reptans | + | + | + |  |  |  | G |
| Alliaria petiolata |  | + |  |  |  |  | F |
| Allium vineale |  | + |  |  |  |  | G |
| Alnus glutinosa | + | + | + |  |  |  | F |
| Alopecurus pratensis |  | + | + |  |  |  | G |
| Amaranthus hybridus |  | + |  |  |  |  | R |
| Amaranthus lividus |  | + |  |  |  |  | R |
| Amaranthus retroflexus |  | + |  |  |  |  | R |
| Ambrosia artemisiifolia |  | + |  |  |  |  | R |
| Anagallis arvensis | + |  |  |  |  |  | R |
| Anemone nemorosa | + |  |  |  |  |  | F |
| Angelica sylvestris/ sylvestris | + | + | + |  |  |  | R |
| Anthoxanthum odoratum |  | + |  |  |  |  | G |
| Anthriscus sylvestris | + |  |  |  |  |  | R |
| Apera spica-venti |  | + |  |  |  |  | R |
| Arctium lappa |  |  | + |  |  |  | R |
| Armoracia rusticana |  |  | + |  |  |  | R |
| Arrhenatherum elatius | + | + | + |  |  |  | G |
| Artemisia vulgaris | + | + | + |  |  |  | R |
| Asarum europaeum agg. | + |  |  |  |  |  | F |
| Aster novae-angliae |  |  | + |  |  |  | R |
| Astragalus glycyphyllos |  |  | + |  |  |  | R |
| Astrantia major/ major |  |  | + |  |  |  | G |
| Athyrium filix-femina | + | + | + |  |  |  | F |
| Bellis perennis | + | + | + |  |  |  | G |
| Betonica officinalis | + | + | + |  |  |  | G |
| Betula pendula | + | + | + |  |  |  | F |
| Bidens frondosa |  |  | + |  |  |  | R |
| Bidens tripartitus |  | + | + |  |  |  | R |
| Bothriochloa ischaemum |  |  | + |  |  |  | R |
| Brachypodium rupestre | + |  |  |  |  |  | G |
| Brachypodium sylvaticum | + | + | + |  |  |  | F |
| Briza media |  | + |  |  |  |  | G |
| Calamagrostis epigejos | + | + | + |  |  |  | R |
| Calluna vulgaris |  |  | + |  |  |  | G |
| Caltha palustris/ palustris | + |  |  |  |  |  | G |
| Calystegia sepium | + | + | + |  |  |  | R |
| Campanula glomerata | + |  |  |  |  |  | G |
| Campanula patula/ patula | + | + |  |  |  |  | G |
| Campanula persicifolia | + | + | + |  |  |  | F |
| Campanula rotundifolia |  | + | + |  |  |  | G |
| Campanula trachelium | + |  |  |  |  |  | F |
| Capsella bursa-pastoris |  | + | + |  |  |  | R |
| Cardamine flexuosa |  | + |  |  |  |  | G |
| Cardamine hirsuta |  | + |  |  |  |  | R |
| Cardamine impatiens |  | + | + |  |  |  | F |
| Carex brizoides | + |  | + |  |  |  | F |
| Carex digitata | + |  |  |  |  |  | F |
| Carex hirta | + | + | + |  |  |  | R |
| Carex montana |  |  | + |  |  |  | F |
| Carex otrubae |  |  | + |  |  |  | G |
| Carex pallescens |  | + |  |  |  |  | G |
| Carex pilosa | + |  |  |  |  |  | F |
| Carex pilulifera |  | + | + |  |  |  | F |
| Carex spicata |  |  | + |  |  |  | G |
| Carex sylvatica | + | + | + |  |  |  | F |
| Carlina acaulis/ acaulis |  |  | + |  |  |  | G |
| Carpinus betulus | + | + | + |  |  |  | F |
| Castanea sativa | + | + | + |  |  |  | F |
| Centaurea carniolica | + | + | + |  |  |  | G |
| Centaurea jacea | + | + | + |  |  |  | G |
| Centaurea pannonica |  | + |  |  |  |  | G |
| Centaurea scabiosa/ scabiosa |  |  | + |  |  |  | G |
| Centaurium erythraea | + | + | + |  |  |  | R |
| Cerastium brachypetalum | + |  |  |  |  |  | R |
| Cerastium glomeratum |  | + | + |  |  |  | R |
| Cerastium holosteoides | + | + | + |  |  |  | R |
| Cerastium sylvaticum | + |  |  |  |  |  | F |
| Chamaecytisus hirsutus |  | + |  |  |  |  | G |
| Chamaecytisus supinus |  | + | + |  |  |  | G |
| Chelidonium majus | + | + | + |  |  |  | R |
| Chenopodium album | + | + | + |  |  |  | R |
| Chenopodium polyspermum |  | + |  |  |  |  | R |
| Chenopodium strictum | + |  |  |  |  |  | R |
| Chrysosplenium alternifolium | + |  |  |  |  |  | F |
| Cichorium intybus | + | + | + |  |  |  | R |
| Circaea lutetiana | + | + | + |  |  |  | F |
| Cirsium arvense | + | + | + |  |  |  | R |
| Cirsium canum |  |  | + |  |  |  | G |
| Cirsium oleraceum | + |  | + |  |  |  | G |
| Cirsium vulgare | + |  | + |  |  |  | R |
| Clematis vitalba | + |  | + |  |  |  | R |
| Clinopodium vulgare/ vulgare | + | + | + |  |  |  | G |
| Colchicum autumnale |  |  | + |  |  |  | G |
| Convallaria majalis | + |  | + |  |  |  | F |
| Convolvulus arvensis | + | + | + |  |  |  | R |
| Conyza canadensis | + | + | + |  |  |  | R |
| Cornus sanguinea agg. | + | + | + |  |  |  | F |
| Corylus avellana | + | + | + |  |  |  | F |
| Crataegus monogyna | + | + | + |  |  |  | F |
| Crepis biennis | + | + | + |  |  |  | R |
| Crepis capillaris | + | + |  |  |  |  | R |
| Cruciata glabra | + | + | + |  |  |  | F |
| Cyclamen purpurascens | + | + | + |  |  |  | F |
| Cynodon dactylon |  | + |  |  |  |  | R |
| Cynosurus cristatus | + |  |  |  |  |  | G |
| Cystopteris fragilis agg. | + | + | + |  |  |  | F |
| Dactylis glomerata/ glomerata | + | + | + |  |  |  | G |
| Dactylis glomerata/ polygama |  |  | + |  |  |  | F |
| Danthonia decumbens |  | + |  |  |  |  | G |
| Daphne mezereum | + | + |  | 1 |  | 1 | F |
| Daucus carota | + | + | + |  |  |  | G |
| Deschampsia caespitosa | + |  | + |  |  |  | G |
| Dianthus armeria/ armeria | + | + | + | 1 |  |  | R |
| Dianthus carthusianorum |  | + |  | 1 |  |  | G |
| Dianthus deltoides |  | + | + | 1 |  |  | G |
| Digitalis grandiflora | + |  |  |  |  |  | F |
| Digitaria sanguinalis/ sanguinalis | + | + | + |  |  |  | R |
| Dryopteris affinis agg. | + |  | + |  |  |  | F |
| Dryopteris carthusiana | + |  | + |  |  |  | F |
| Dryopteris dilatata | + |  |  |  |  |  | F |
| Dryopteris filix-mas | + | + | + |  |  |  | F |
| Echinochloa crus-galli | + | + | + |  |  |  | R |
| Elytrigia repens | + | + | + |  |  |  | R |
| Epilobium montanum | + | + | + |  |  |  | F |
| Epilobium parviflorum |  | + | + |  |  |  | R |
| Epilobium tetragonum/ tetragonum |  | + | + |  |  |  | R |
| Epipactis helleborine agg. |  | + | + | 1 |  |  | F |
| Equisetum arvense | + | + | + |  |  |  | R |
| Equisetum palustre |  |  | + |  |  |  | R |
| Equisetum pratense | + |  | + |  | 1 |  | F |
| Equisetum sylvaticum | + | + | + |  |  |  | F |
| Equisetum telmateia | + | + | + |  |  |  | F |
| Erechtites hieraciifolia | + |  | + |  |  |  | R |
| Erigeron annuus/ annuus | + | + | + |  |  |  | R |
| Euonymus europaea | + | + | + |  |  |  | F |
| Eupatorium cannabinum | + | + | + |  |  |  | R |
| Euphorbia amygdaloides | + |  |  |  |  |  | F |
| Euphorbia cyparissias | + | + | + |  |  |  | R |
| Euphorbia dulcis | + | + | + |  |  |  | R |
| Euphorbia stricta |  | + |  |  |  |  | R |
| Euphrasia rostkoviana |  | + | + |  |  |  | G |
| Fagus sylvatica | + | + | + |  |  |  | F |
| Fallopia convolvulus | + |  |  |  |  |  | R |
| Festuca gigantea | + |  | + |  |  |  | F |
| Festuca pratensis |  | + | + |  |  |  | G |
| Festuca rubra agg. |  | + | + |  |  |  | G |
| Festuca rupicola |  |  | + |  |  |  | G |
| Filaginella uliginosa |  | + | + |  |  |  | R |
| Filipendula ulmaria/ ulmaria |  | + | + |  |  |  | G |
| Fragaria vesca | + | + | + |  |  |  | F |
| Frangula alnus | + | + | + |  |  |  | F |
| Fraxinus excelsior | + |  | + |  |  |  | F |
| Galeobdolon montanum | + | + | + |  |  |  | F |
| Galeopsis pubescens | + | + | + |  |  |  | F |
| Galeopsis speciosa | + | + | + |  |  |  | R |
| Galinsoga ciliata | + | + |  |  |  |  | R |
| Galinsoga parviflora |  | + | + |  |  |  | R |
| Galium aparine | + | + | + |  |  |  | R |
| Galium mollugo | + | + | + |  |  |  | G |
| Galium odoratum | + | + | + |  |  |  | F |
| Galium rotundifolium | + | + | + |  |  |  | F |
| Galium sylvaticum | + | + | + |  |  |  | F |
| Galium verum |  | + | + |  |  |  | G |
| Genista tinctoria |  |  | + |  |  |  | F |
| Gentiana asclepiadea | + | + | + | 1 |  | 1 | F |
| Gentiana pneumonanthe |  |  | + | 1 |  | 1 | G |
| Geranium columbinum | + |  |  |  |  |  | R |
| Geranium palustre |  | + |  |  |  | 1 | G |
| Geranium pusillum |  |  | + |  |  |  | R |
| Geranium robertianum |  | + | + |  |  |  | R |
| Geum urbanum | + | + | + |  |  |  | F |
| Glechoma hederacea | + | + | + |  |  |  | F |
| Hedera helix | + | + | + |  |  |  | F |
| Helianthemum nummularium |  |  | + | 1 |  | 1 | G |
| Helianthus annuus |  | + |  |  |  |  | R |
| Helictotrichon pubescens |  | + |  |  |  |  | G |
| Heracleum sphondyllium/ sphon. | + | + | + |  |  |  | R |
| Hieracium lactucella |  | + | + |  |  | 1 | G |
| Hieracium murorum | + | + | + |  |  |  | F |
| Hieracium pilosella | + | + | + |  |  |  | G |
| Hieracium praealtum |  | + | + |  |  |  | G |
| Hieracium racemosum | + | + | + |  |  |  | F |
| Hieracium sabaudum | + | + |  |  |  |  | F |
| Hieracium umbellatum | + | + | + |  |  |  | G |
| Holcus lanatus | + | + | + |  |  |  | G |
| Holcus mollis |  | + |  |  |  | 1 | G |
| Humulus lupulus | + | + | + |  |  |  | R |
| Hypericum humifusum |  |  | + |  |  |  | R |
| Hypericum perforatum | + | + | + |  |  |  | R |
| Hypochoeris radicata | + | + | + |  |  |  | G |
| Impatiens glandulifera | + |  | + |  |  |  | R |
| Impatiens noli-tangere | + | + | + |  |  |  | R |
| Impatiens parviflora | + |  |  |  |  |  | R |
| Juglans regia | + | + | + |  |  |  | F |
| Juncus articulatus |  | + |  |  |  |  | R |
| Juncus bufonius |  | + |  |  |  |  | R |
| Juncus compressus | + | + | + |  |  |  | R |
| Juncus effusus | + | + | + |  |  |  | G |
| Juncus tenuis |  |  | + |  |  |  | R |
| Juniperus communis |  | + |  |  |  |  | F |
| Knautia arvensis | + | + | + |  |  |  | G |
| Knautia drymeia/ drymeia | + | + | + |  |  |  | F |
| Lactuca serriola | + |  |  |  |  |  | R |
| Lapsana communis |  |  | + |  |  |  | F |
| Lathyrus pratensis | + | + | + |  |  |  | G |
| Lathyrus sylvaticus |  | + |  |  |  |  | F |
| Lathyrus vernus/ vernus |  |  | + |  |  |  | F |
| Leersia oryzoides |  | + |  |  |  |  | R |
| Lembotropis nigricans/ nigricans | + | + | + |  |  |  | F |
| Leontodon autumnalis | + | + | + |  |  |  | G |
| Leontodon hispidus/ danubialis | + | + | + |  |  |  | G |
| Leontodon hispidus/ hispidus |  | + | + |  |  |  | G |
| Leucanthemum ircutianum |  | + | + |  |  |  | G |
| Leucanthemum praecox |  | + |  |  |  |  | G |
| Ligustrum vulgare | + | + | + |  |  |  | F |
| Linaria vulgaris | + |  |  |  |  |  | R |
| Linum catharticum/ catharticum |  |  | + |  |  |  | G |
| Lolium multiflorum |  | + |  |  |  |  | R |
| Lolium perenne | + | + | + |  |  |  | G |
| Lotus corniculatus/ corniculatus | + | + | + |  |  |  | G |
| Luzula luzuloides | + | + | + |  |  |  | F |
| Luzula multiflora |  | + |  |  |  |  | F |
| Luzula pilosa | + | + | + |  |  |  | F |
| Lychnis flos-cuculi |  | + |  |  |  |  | G |
| Lycopus europaeus/ europaeus | + | + | + |  |  |  | R |
| Lysimachia nummularia | + | + | + |  |  |  | G |
| Lysimachia punctata | + | + | + |  |  |  | R |
| Lysimachia vulgaris | + | + | + |  |  |  | G |
| Lythrum salicaria | + | + | + |  |  |  | G |
| Maianthemum bifolium | + | + | + |  |  |  | F |
| Malus domestica | + |  |  |  |  |  | F |
| Malus sylvsetris |  |  | + |  |  |  | F |
| Malva alcea |  |  | + |  |  |  | R |
| Medicago lupulina | + |  |  |  |  |  | R |
| Medicago sativa | + | + |  |  |  |  | R |
| Melampyrum pratense | + | + | + |  |  |  | F |
| Melica nutans |  | + |  |  |  |  | F |
| Melittis melissophyllum | + | + | + |  |  |  | F |
| Mentha arvensis/ arvensis | + |  | + |  |  |  | R |
| Moehringia trinervia |  | + | + |  |  |  | F |
| Molinia arundinacea |  |  | + |  |  |  | G |
| Mycelis muralis | + | + | + |  |  |  | F |
| Myosotis arvensis | + | + | + |  |  |  | R |
| Myosotis scorpioides |  | + | + |  |  |  | G |
| Myosoton aquaticum | + |  |  |  |  |  | F |
| Neottia nidus-avis | + | + | + | 1 |  |  | F |
| Odontites vernus/ vernus |  |  | + | 1 |  | 1 | G |
| Ononis spinosa |  | + |  |  |  |  | G |
| Origanum vulgare/ vulgare |  |  | + |  |  |  | G |
| Oxalis acetosella | + | + | + |  |  |  | F |
| Oxalis fontana | + | + | + |  |  |  | R |
| Panicum dichotomiflorum | + |  |  |  |  |  | R |
| Panicum miliaceum agg. | + |  |  |  |  |  | R |
| Parthenocissus inserta | + |  |  |  |  |  | F |
| Pastinaca sativa/ sativa | + | + | + |  |  |  | R |
| Peucedanum oreoselinum | + | + | + |  |  |  | G |
| Phegopteris connectilis | + |  |  |  |  |  | F |
| Phragmites australis | + |  |  |  |  |  | G |
| Picea abies | + | + | + |  |  |  | F |
| Picea pungens |  |  | + |  |  |  | F |
| Picris hieraciioides | + | + | + |  |  |  | R |
| Pimpinella major/ major |  | + | + |  |  |  | G |
| Pimpinella saxifraga/ saxifraga | + | + | + |  |  |  | G |
| Pinus sylvestris | + | + | + |  |  |  | F |
| Plantago intermedia |  | + | + |  |  |  | R |
| Plantago lanceolata | + | + | + |  |  |  | G |
| Plantago major | + | + | + |  |  |  | R |
| Plantago media | + | + | + |  |  |  | G |
| Platanthera bifolia | + | + |  | 1 |  |  | F |
| Poa annua | + | + | + |  |  |  | R |
| Poa compressa |  | + |  |  |  |  | R |
| Poa nemoralis | + | + | + |  |  |  | F |
| Poa pratensis | + |  |  |  |  |  | G |
| Poa trivialis | + |  |  |  |  |  | F |
| Polygala comosa |  | + | + |  |  |  | G |
| Polygala vulgaris/ vulgaris |  | + | + |  |  |  | G |
| Polygonatum multiflorum | + |  | + |  |  |  | F |
| Polygonum aviculare/ aviculare | + | + | + |  |  |  | R |
| Polygonum hydropiper |  | + | + |  |  |  | R |
| Polygonum lapathifolium/ lapathifolium | + | + | + |  |  |  | R |
| Polygonum minus |  |  | + |  |  |  | R |
| Polygonum mite | + | + | + |  |  |  | R |
| Polygonum persicaria | + | + | + |  |  |  | R |
| Polypodium vulgare |  | + |  |  |  |  | F |
| Populus tremula | + | + | + |  |  |  | F |
| Potentilla alba |  |  | + |  |  |  | G |
| Potentilla anserina | + |  |  |  |  |  | R |
| Potentilla erecta | + | + | + |  |  |  | G |
| Potentilla reptans | + | + | + |  |  |  | R |
| Prenanthes purpurea | + | + | + |  |  |  | F |
| Primula vulgaris | + | + | + | 1 |  |  | G |
| Prunella laciniata |  | + |  | 1 |  |  | G |
| Prunella vulgaris | + | + | + |  |  |  | G |
| Prunus avium | + | + | + |  |  |  | F |
| Prunus domestica | + | + | + |  |  |  | F |
| Prunus padus | + | + | + |  |  |  | F |
| Prunus spinosa | + | + | + |  |  |  | F |
| Pseudolysimachion orchideum |  |  | + | 1 | 1 |  | G |
| Pteridium aquilinum | + | + | + |  |  |  | F |
| Pulmonaria officinalis | + | + | + |  |  |  | F |
| Pyrus pyraster | + | + | + |  |  |  | F |
| Quercus petraea | + | + | + |  |  |  | F |
| Quercus robur | + | + | + |  |  |  | F |
| Ranunculus acris/ acris | + | + | + |  |  |  | G |
| Ranunculus arvensis |  |  | + |  |  |  | R |
| Ranunculus bulbosus |  | + | + |  |  |  | G |
| Ranunculus nemorosus |  | + | + |  |  | 1 | F |
| Ranunculus repens | + | + | + |  |  |  | R |
| Ranunculus sardous |  | + | + |  |  |  | R |
| Rhamnus catharticus |  |  | + |  |  |  | F |
| Rhus typhina |  | + |  |  |  |  | R |
| Robinia pseudacacia | + | + | + |  |  |  | R |
| Rorippa sylvatica |  |  | + |  |  |  | R |
| Rosa canina agg. | + | + | + |  |  |  | R |
| Rosa tomentosa | + |  |  |  |  | 1 | R |
| Rubus caesius agg. |  |  | + |  |  |  | R |
| Rubus hirtus agg. | + | + | + |  |  |  | F |
| Rubus idaeus | + | + | + |  |  |  | R |
| Rubus sulcatus agg. | + | + | + |  |  |  | R |
| Rumex acetosa |  | + | + |  |  |  | G |
| Rumex acetosella/ acetosella |  | + | + |  |  |  | R |
| Rumex crispus | + |  | + |  |  |  | R |
| Rumex obtusifolius/ obtusifolius | + | + | + |  |  |  | R |
| Rumex sanguineus |  | + |  |  |  |  | F |
| Salix alba |  | + |  |  |  |  | F |
| Salix aurita |  | + |  |  |  |  | R |
| Salix caprea | + | + | + |  |  |  | R |
| Salix cinerea | + | + |  |  |  |  | R |
| Salvia glutinosa | + | + | + |  |  |  | F |
| Salvia pratensis |  | + |  |  |  |  | G |
| Sambucus ebulus |  | + | + |  |  |  | R |
| Sambucus nigra | + | + | + |  |  |  | R |
| Sambucus racemosa | + |  |  |  |  | 1 | F |
| Sanguisorba officinalis | + | + | + |  |  |  | G |
| Sanicula europaea | + | + | + |  |  |  | F |
| Scabiosa triandra |  | + | + |  |  |  | G |
| Scirpus sylvaticus | + | + | + |  |  |  | G |
| Scrophularia nodosa | + | + |  |  |  |  | R |
| Scutellaria galericulata | + |  |  |  |  |  | G |
| Sedum maximum | + |  |  |  |  |  | F |
| Selinum carvifolia | + | + | + |  |  |  | R |
| Senecio ovatus | + |  |  |  |  |  | F |
| Serratula tinctoria/ tinctoria | + |  |  |  |  |  | G |
| Seseli annuum |  |  | + |  |  |  | G |
| Setaria pumila | + | + | + |  |  |  | R |
| Silene latifolia/ latifolia |  |  | + |  |  |  | R |
| Solanum dulcamara | + | + | + |  |  |  | R |
| Solanum nigrum/ nigrum |  |  | + |  |  |  | R |
| Solidago gigantea | + | + | + |  |  |  | R |
| Solidago virgaurea/ virgaurea | + | + | + |  |  |  | F |
| Sonchus arvensis |  | + | + |  |  |  | R |
| Sonchus asper | + |  | + |  |  |  | R |
| Sorbus domestica |  | + |  | 1 |  | 1 | F |
| Sorbus torminalis | + |  | + |  |  |  | F |
| Spergula arvensis/ arvensis |  | + |  |  |  |  | R |
| Stachys palustris |  |  | + |  |  |  | R |
| Stachys sylvatica |  | + |  |  |  |  | F |
| Stellaria graminea | + | + | + |  |  |  | G |
| Stellaria holostea | + |  | + |  |  |  | F |
| Stellaria media |  |  | + |  |  |  | R |
| Stellaria neglecta | + | + | + |  |  |  | F |
| Succisa pratensis |  |  | + |  |  |  | G |
| Succisella inflexa |  |  | + | 1 | 1 | 1 | G |
| Symphytum officinale/ officinale | + |  | + |  |  |  | R |
| Tanacetum vulgare | + | + | + |  |  |  | R |
| Taraxacum officinale agg. | + | + | + |  |  |  | G |
| Thalictrum lucidum |  | + |  |  |  |  | R |
| Thesium linophyllon |  |  | + |  |  |  | G |
| Thuja occidentalis | + |  |  |  |  |  | F |
| Thymus pulegioides/ pulegioides | + | + | + |  |  |  | G |
| Tilia cordata | + | + | + |  |  |  | F |
| Tilia platyphyllos | + |  | + |  |  |  | F |
| Torilis japonica | + | + | + |  |  |  | R |
| Tragopogon pratensis/ orientalis |  | + | + |  |  |  | G |
| Trifolium alpestre | + | + | + |  |  |  | G |
| Trifolium dubium |  | + |  |  |  |  | G |
| Trifolium incarnatum |  | + |  |  |  |  | R |
| Trifolium pratense/ pratense | + | + | + |  |  |  | G |
| Trifolium repens | + | + | + |  |  |  | G |
| Trisetum flavescens | + | + | + |  |  |  | G |
| Tussilago farfara | + | + | + |  |  |  | R |
| Ulmus minor | + |  |  |  |  |  | F |
| Urtica dioica | + | + | + |  |  |  | R |
| Vaccinium myrtillus | + |  | + |  |  |  | F |
| Verbascum blattaria | + |  |  |  |  |  | R |
| Veronica chamaedrys | + | + | + |  |  |  | R |
| Veronica officinalis | + | + | + |  |  |  | F |
| Veronica persica |  |  | + |  |  |  | R |
| Veronica polita | + |  |  |  |  |  | R |
| Viburnum opulus | + | + | + |  |  |  | F |
| Vicia cassubica |  |  | + | 1 |  |  | R |
| Vicia cracca | + |  | + |  |  |  | G |
| Vicia sepium | + |  | + |  |  |  | R |
| Vicia tetrasperma |  |  | + |  |  |  | R |
| Vincetoxicum hirundinaria/ hirundinaria |  |  | + |  |  |  | G |
| Viola arvensis |  | + |  |  |  |  | R |
| Viola canina agg. | + | + | + |  |  |  | G |
| Viola hirta |  |  | + |  |  |  | G |
| Viola odorata | + | + |  |  |  |  | F |
| Viola reichenbachiana | + | + | + |  |  |  | F |
| Viola riviniana | + | + | + |  |  |  | F |
| Viscum album | + |  |  |  |  |  | F |
| Vitis vinifera | + |  |  |  |  |  | R |
| Total number of taxa | 281 | 256 | 299 | 3 | 18 | 14 |  |

| Bird species lists |  |  |  |  |  |  |  |
| --- | --- | --- | --- | --- | --- | --- | --- |
| Latin name | English name | AUS | SLO | HUN | Habitat | Abundance | Breeding |
| Accipiter nisus | Sparrowhawk |  | + |  | M | R | rB |
| Aegithalos caudatus | Long-tailed tit | + | + | + | M | C | rB |
| Buteo buteo | Common buzzard | + | + | + | M | C | rB |
| Carduelis carduelis | Goldfinch | + | + | + | M | C | rB |
| Certhia familiaris | Treecreeper | + | + | + | F | C | rB |
| Chloris chloris | Greenfinch | + | + | + | M | vC | rB |
| Coccothraustes coccothraustes | Hawfinch | + | + | + | F | vC | rB |
| Columba oenas | Stock dove | + | + | + | F | C | mB |
| Columba palumbus | Wood pigeon | + | + | + | F | C | rB |
| Corvus corax | Raven | + | + | + | F | C | rB |
| Corvus corone cornix | Hooded crow | + | + | + | M | vC | rB |
| Corvus monedula | Jackdaw | + |  |  | S | R | rB? |
| Coturnix coturnix | Quail |  | + |  | O | R | mB |
| Cuculus canorus | Cuckoo | + | + | + | F | C | mB |
| Cyanistes caeruleus | Blue tit | + | + | + | F | vC | rB |
| Dendrocopos major | Great spotted woodpecker | + | + | + | F | vC | rB |
| Dendrocopos minor | Lesser spotted woodpecker | + | + | + | F | C | rB |
| Dryocopus martius | Black woodpecker | + | + | + | F | C | rB |
| Emberiza citrinella | Yellowhammer | + | + | + | O | vC | rB |
| Erithacus rubecula | Robin | + | + | + | F | vC | rB |
| Falco tinnunculus | Kestrel | + | + |  | O | R | rB |
| Fringila coelebs | Chaffinch | + | + | + | F | vC | rB |
| Garrulus glandarius | Jay | + | + | + | F | vC | rB |
| Hirundo rustica | Barn swallow | + | + | + | S | C | mB |
| Jynx torquilla | Wryneck |  | + | + | O | R | mB |
| Lanius collurio | Red-backed shrike |  | + |  | O | R | mB |
| Lophophanes cristatus | Crested tit |  | + |  | F | R | rB? |
| Merops apiaster | Bee-eater |  |  | + | * | * | * |
| Motacilla alba | White wagtail | + | + | + | S | C | rB |
| Oriolus oriolus | Golden oriole | + | + |  | F | vC | mB |
| Parus major | Great tit | + | + | + | F | vC | rB |
| Passer domesticus | House sparrow | + |  | + | S | C | rB |
| Passer montanus | Tree sparrow | + | + | + | S | vC | rB |
| Phasianus colchicus | Pheasant | + | + | + | M | C | int |
| Phoenicurus ochruros | Black redstart | + | + | + | S | C | rB |
| Phylloscopus collybita | Chiffchaff | + | + | + | F | vC | mB/ rB |
| Picus canus | Grey-headed woodpecker |  | + |  | F | R | rB |
| Picus viridis | Green woodpecker | + | + | + | F | C | rB |
| Poecile palustris | Marsh tit | + | + | + | F | C | rB |
| Pyrrhula pyrrhula | Bullfinch |  |  | + | F | R | rB? |
| Regulus regulus | Goldcrest | + | + |  | F | C | rB |
| Saxicola torquatus | Stonechat |  | + | + | O | C | mB |
| Serinus serinus | Serin | + |  | + | S | C | rB |
| Sitta europaea | Nuthatch | + | + | + | F | vC | rB |
| Streptopelia decaocto | Collared dove | + | + |  | S | C | rB |
| Streptopelia turtur | Turtle dove |  | + |  | O | R | mB |
| Strix aluco | Tawny owl | + |  | + | F | C | rB |
| Sturnus vulgaris | Starling | + | + | + | M | vC | mB |
| Sylvia atricapilla | Blackcap | + | + | + | F | vC | mB |
| Troglodytes troglodytes | Wren | + | + | + | F | vC | rB |
| Turdus merula | Blackbird | + | + | + | F | vC | rB |
| Turdus philomelus | Song thrush | + | + | + | F | vC | rB |
| Turdus viscivorus | Mistle thrush | + | + | + | F | C | rB |

References for supporting information
